# Left ventricular myocardial deformation measurements by magnetic resonance Tissue Tracking agrees with tagging (HARP) in healthy volunteers

**DOI:** 10.1186/1532-429X-18-S1-Q54

**Published:** 2016-01-27

**Authors:** Kanae Mukai, Kimberly Kallianos, Florent Seguro, Gabriel Acevedo-Bolton, Karen Ordovas

**Affiliations:** 1 grid.266102.10000000122976811Radiology and Biomedical Imaging, University of California, San Francisco, San Francisco, CA USA; 2 grid.266102.10000000122976811Cardiology, University of California, San Francisco, San Francisco, CA USA; 3 Cardiology, Federation Francaise de Cardiologie, Toulouse, France

## Background

Left ventricular myocardial strain has been described as a potentially useful technique for evaluation and risk stratification of a range of acquired and congenital heart diseases. Tagged acquisition by cardiac MRI has been considered the reference standard for determining myocardial strain; however, this method can be time consuming and the tagged lines may not be visible throughout the cardiac cycle. Tissue Tracking software (Circle Cardiovascular Imaging®) is a post-processing method which allows measurements of myocardial strain based on cine cardiovascular magnetic resonance images without the need for additional sequences. Augustine et al. (JCMR 2013) have previously reported reasonable agreement between Feature Tracking (Tom Tec®) measurements for circumferential strain and tagging. We aimed to perform a similar comparison between Tissue Tracking software and tagging on a small cohort of normal volunteers.

## Methods

Cardiac MRI studies were performed with a 1.5 T unit (Achieva, Philips Medical Systems) on 7 normal volunteers. Short-axis tagged MR images of the left ventricle were analyzed using HARP software (Diagnosoft, Cary, NC) by the same observer, blinded to results of the alternate method. Steady state free precession (SSFP) cine images were analyzed with Tissue Tracking software (Circle Cardiovascular Imaging®) by two observers in consensus. The 2D peak circumferential strain (%) obtained by both techniques were compared using Bland Altman analysis for each of the 16 cardiac segments based on the American Heart Association classification for short axis images.

## Results

**Figure 1:**
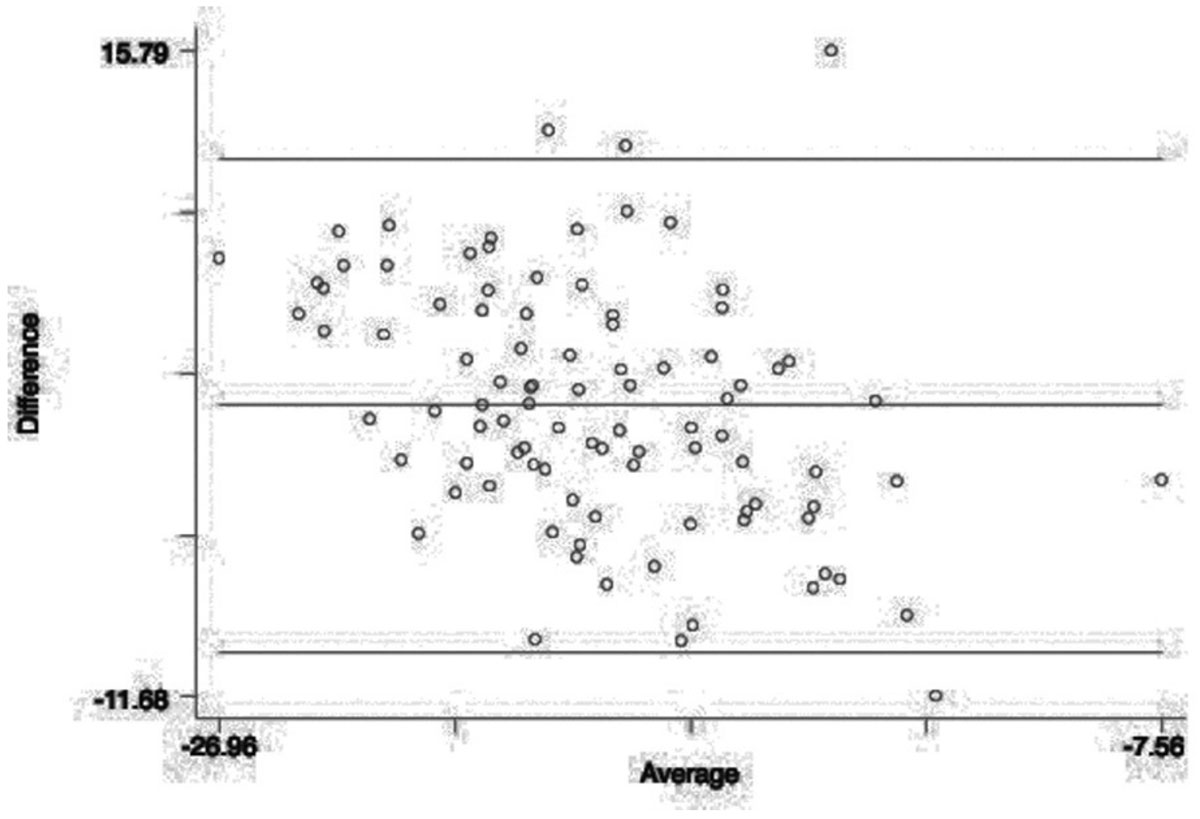
**Bland Altman plot comparing Tissue Tracking and CMR tagging (HARP) for 2D peak circumferential strain (%) of the left ventricle**.

There was reasonably good correlation between tagged acquisition and Tissue Tracking software. A total of 94 data points were included. The Bland-Altman plot is shown in Figure 1. The limits of agreement or reference range for difference was -9.780% to 11.222% with a mean difference of 0.721% (CI -0.355 to 1.796). The range was - 26.960% to -7.560%. The Pitman's test of difference in variance suggested overall concordance with r = -0.391 (n = 94, p = 0.001).

## Conclusions

Tissue Tracking software provides a good surrogate for calculating 2D peak circumferential strain (%). A larger cohort is needed to further validate this new capability.

